# Bat Influenza Viruses: Current Status and Perspective

**DOI:** 10.3390/v13040547

**Published:** 2021-03-25

**Authors:** Wenyu Yang, Tony Schountz, Wenjun Ma

**Affiliations:** 1 Department of Veterinary Pathobiology, College of Veterinary Medicine, University of Missouri, Columbia, MO 65211, USA; wymk2@missouri.edu; 2 Department of Molecular Microbiology and Immunology, School of Medicine, University of Missouri, Columbia, MO 65211, USA; 3 Center for Vector-Borne Infectious Diseases, Department of Microbiology, Immunology and Pathology, College of Veterinary Medicine and Biomedical Sciences, Colorado State University, Fort Collins, CO 80523, USA; Tony.Schountz@colostate.edu

**Keywords:** bat influenza viruses, virus replication in vitro and in vivo, zoonotic potential, perspective

## Abstract

Bats are natural reservoirs for many viruses, including several that are zoonotic. Two unusual H17N10 and H18N11 influenza viruses have been found in New World bats. Although neither of these viruses have been isolated, infectious clone technology has permitted significant progress to understand their biology, which include unique features compared to all other known influenza A viruses. In addition, an H9N2-like influenza A virus was isolated from Old World bats and it shows similar characteristics of normal influenza A viruses. In this review, current status and perspective on influenza A viruses identified in bats is reviewed and discussed.

## 1. Introduction

Influenza is an important respiratory disease that is responsible for more than 30,000 deaths each year during seasonal epidemics in the USA alone [1,2,3]. The disease is principally caused by influenza A virus (IAV) and influenza B virus (IBV) that belong to the *Orthomyxoviridae* family. IAV is an enveloped, negative-stranded RNA virus with eight gene segments that readily acquire mutations under host immune pressure and undergo reassortment with different strains and subtypes [4,5]. IAV is a zoonotic pathogen that can infect a broad range of species, such as birds and mammals. Waterfowl and shore birds have been considered to be natural reservoirs of IAVs, as all H1–H16 subtypes have been isolated from them and routinely circulate in these birds [6,7,8]. However, only certain subtypes of IAVs establish and routinely circulate in mammalian species such as H1N1 and H3N2 subtypes in humans, H1N1, H1N2, and H3N2 subtypes in swine [6], and H3N8 and H7N7 subtypes in horses [9,10]. The receptors used by IAVs are some of several major factors that serve as species barriers. For example, avian influenza viruses preferentially bind to α-2,3 sialic acids, whereas human influenza viruses preferentially bind to α-2,6 sialic acids [11,12]. IAVs from avian species typically do not spill over to humans, although it sometimes occurs with H5, H7, and H9 subtypes of human infections that have been documented to have a potential to become pandemic [13,14,15,16,17,18,19]. Noticeably, IAV has bidirectional transmission between human and swine [20], which is exemplified by swine-origin 2009 pandemic H1N1 [21,22,23]. Since the detection of genome sequences and isolation of IAVs have been identified from bat samples, the host species of IAVs has been expanded to bats [24,25,26,27]. In contrast to known H1–H16 IAVs, both H17N10 and H18N11 viruses are distinct from conventional IAVs in multiple aspects, such as virus cultivation and replication, cellular receptors used and neuraminidase (NA) functions, although they have similar genomes and are close to IAVs [24,25]. In addition, a novel H9N2-like IAV was isolated from Egyptian fruit bats (*Rousettus aegyptiacus*), which is also distant from known conventional IAVs [27]. To date, both H17N10 and H18N11, as well as the novel H9N2-like virus, have only been found in bats, suggesting that bats could be natural reservoirs of distinct IAVs. In this review, we will summarize the current status of bat influenza viruses and discuss future studies that should clarify their biology.

## 2. Discovery of Bat Influenza Viruses

### 2.1. H17N10 Bat Influenza Virus

**Figure 1:**
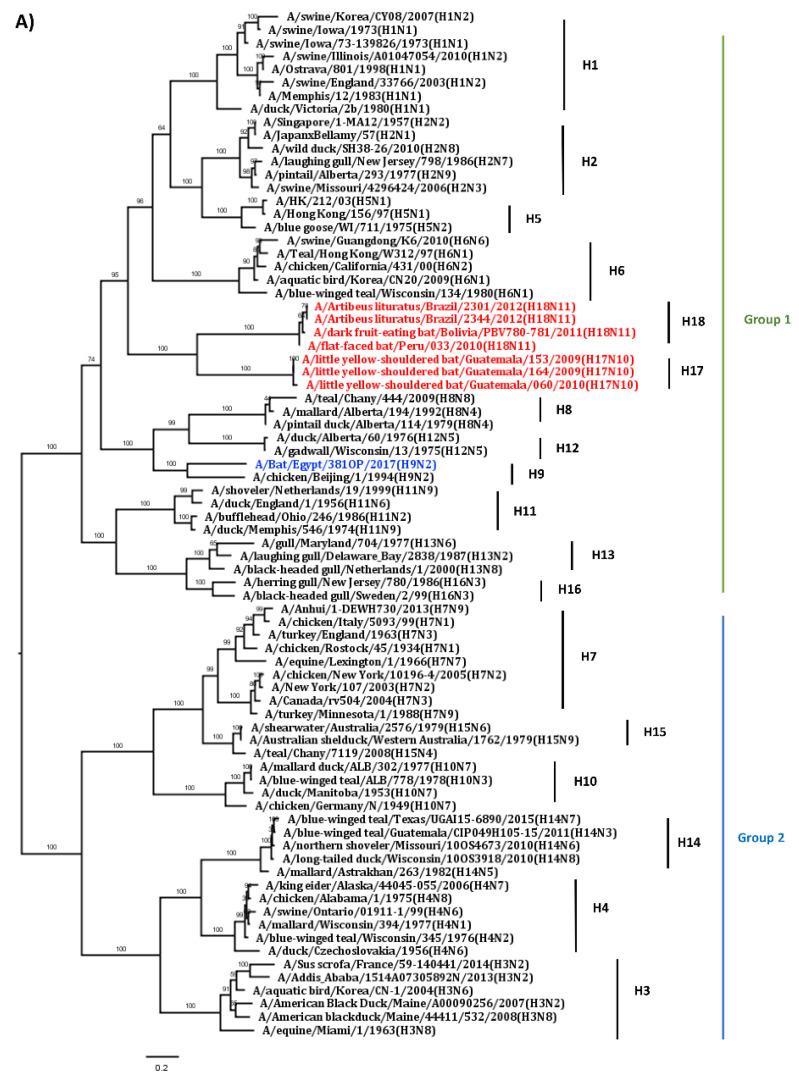

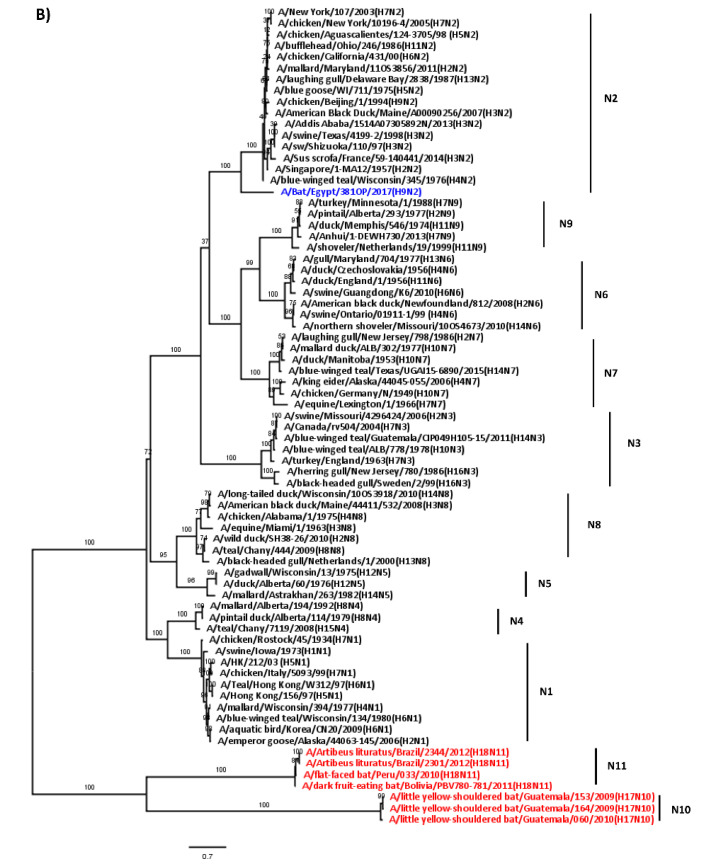

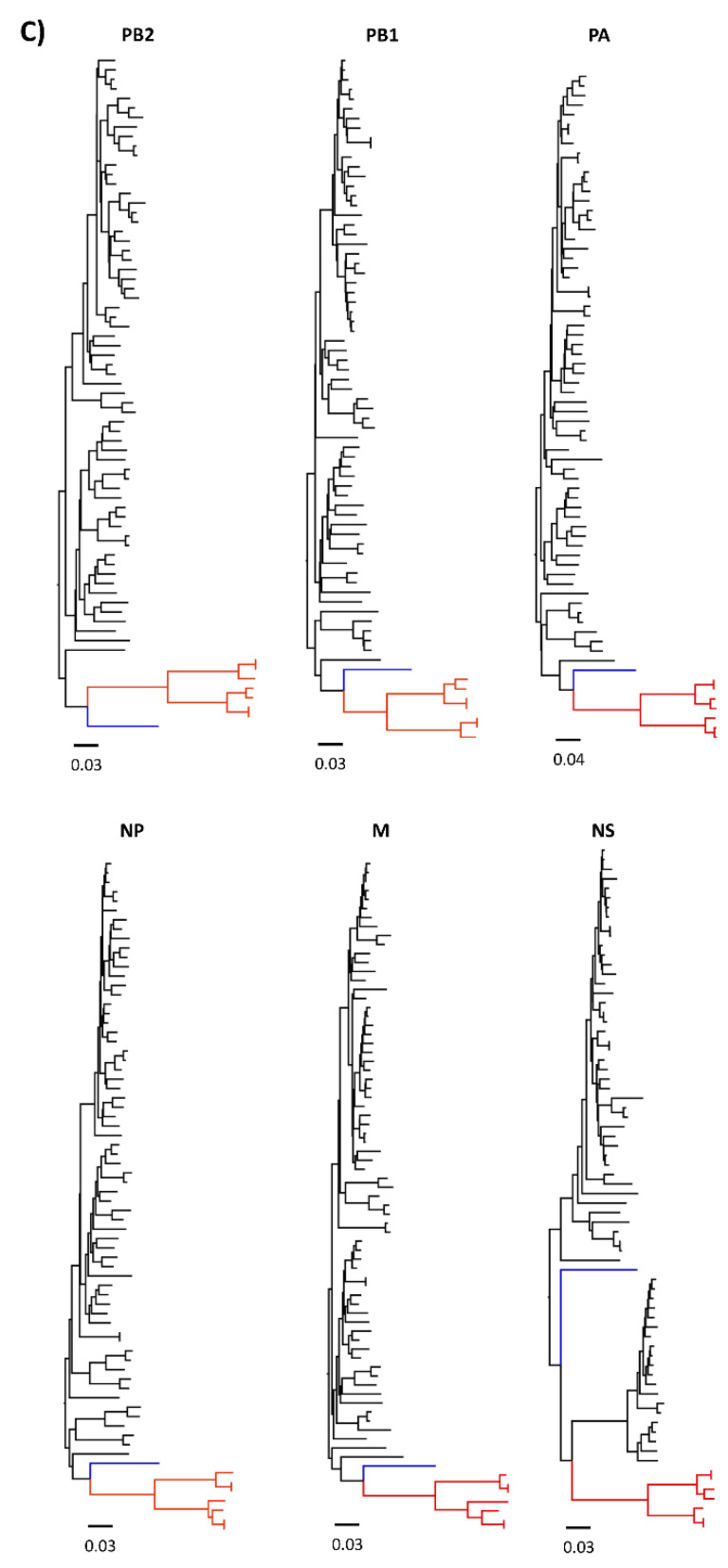
Phylogenetic trees of bat influenza virus genes. (**A**) Phylogenetic tree of HA gene. (**B**) Phylogenetic tree of NA gene. (**C**) Phylogenetic trees of internal genes. We downloaded 70 full-length genome sequences from different subtypes of conventional influenza A viruses (IAVs) and eight bat influenza viruses, including 3 H17N10, 4 H18N11 and 1 H9N2-like bat viruses from public database. Phylogenetic analyses for each gene were performed based on their full-length gene sequences. The sequences were aligned by the MUSCLE method by MegAlign Pro 17. Phylogenetic relationships among bat HA and NA sequences with those from conventional IAVs were calculated using the maximum likelihood method by MegAlign Pro 17, while for the internal genes determined by using the Neighbor joining method by MegAlign Pro 17. Phylogenetic trees were visualized in FigTree (v1.4.3; http://tree.bio.ed.ac.uk/software/figtree/). Red color represents bat H17N10 and H18N11 viruses; blue color represents A/Bat/Egypt/381OP/2017 (H9N2) virus.

In 2009–2010, a novel influenza virus was discovered by pan-rtPCR and sequencing, and its full-genome sequence was then determined by next-generation sequencing from samples collected from New World yellow-shouldered fruit bats (*Sturnira lilium*) in Guatemala [24]. Although the identified viral genome conforms to the characteristics of IAV genomes and each gene segment is a close match with those of the IAVs, they were phylogenetically distinct from those of known IAVs (Figure 1) [24,28]. Analysis of the hemagglutinin (HA) genes suggests that the HA gene is closer to those in the Group 1 (H1, H2, H5, H6, H8, H9, H11, H12, H13, and H16) than those in the Group 2 (H3, H4, H7, H10, H14, and H15) and forms a separate branch (Figure 1A) [29].

Regarding the NA gene of the bat virus, it is substantially divergent from IAV branches and forms a separate branch (Figure 1B), and they might have diverged from ancient IAVs. Because the key structural features of its genome are consistent with those of conventional IAVs, it is classified as an IAV. In addition, the amino acid sequence of its HA has only 45% identity with those of conventional IAVs, whereas its NA shows 24% identity with those of conventional IAVs. Based on significant difference from all known IAV subtypes, the novel virus (A/bat/Guat/09) has been classified as an H17N10 subtype [24]. Evolutionary analysis suggests that the H17N10 bat influenza virus is ancestral to currently known conventional IAVs [30].

To date, three H17N10 genome sequences have been detected in bat samples in Guatemala; two were found in samples collected in EI Jobo in 2009 and one in samples collected during 2010 in Aguero that is approximately 50 km from EI Jobo [24]. Interestingly, these three H17N10 genome sequences are similar and show high homology in each gene segment [24]. Detection of the H17N10 genome sequences in different places and time suggests that the H17N10 virus has been circulating in bats for many years.

### 2.2. H18N11 Bat Influenza Virus

In 2010, H18N11 sequences were first detected in rectal swabs collected from New World flat-faced fruit bats (*Artibeus planirostris*) in Peru [25]. Subsequently, the H18N11 genome sequences were also found in dark fruit-eating bats (*Artibeus obscurus*) in Bolivia in 2015 (A/dark fruit-eating bat/Bolivia/PNV780-781/2011 (H18N11)) and in great fruit bats (*Artibeus lituratus*) from Brazil in 2019 [26]. Sequence and phylogenic analysis show that eight gene segments are divergent from those of conventional IAVs and are most close to those of the H17N10 virus but form separate branches [25,26]. Phylogenic analysis reveals that the NA and internal genes of the H18N11 virus cannot be classified into any groups of conventional IAVs and are different from those of the H17N10 virus (Figure 1B and C). Unlike NA and internal genes, the HA gene of the H18N11 virus also belongs to the Group 1 of IAVs according to the phylogenetic analysis (Figure 1A) [25,26,29]. Although both H18N11 and H17N10 are of bat-origin and share phylogenetic proximity, their gene and protein sequences display low identity, ranging from 48.7% to 62.3% at the nucleotide level and 76.2% to 81.6% at amino acid level [25], leading to classification of H18N11 as a distinct subtype.

Both Peruvian and Brazilian H18N11 genome sequences were only detected in intestines not in other tissue samples such as liver and spleen from positive bats [25,26,29], suggesting that the virus likely is confined to the gastrointestinal tract of bats. Furthermore, the genome sequences of two Brazilian H18N11 viruses show high identities with that of the Peruvian H18N11 virus [26]. Peruvian and Brazilian H18N11 genome sequences have been found in different locations (>2000 km) and in different bat species, suggesting that H18N11 virus has been circulating among bats for many years. Given the phylogenetic distance between H17N10 and H18N11, it may be that many other bat IAVs are circulating in New World bats, or that their distinct reservoir host species have imposed substantial pressure on their genomes during host adaptation that has led to greater genetic differences between the viruses. In support of this latter hypothesis, it has been 10 years since these viruses were discovered, yet no other similar viruses have been discovered.

### 2.3. H9N2-Like Bat Influenza Virus

A novel H9N2-like virus (A/bat/Egypt/381OP/2017) was detected in oral and fecal swab samples collected from Old World Egyptian fruit bats in densely populated agricultural area in 2017 in Egypt [27]. This virus was isolated using chicken eggs and replicated very efficiently in Madin–Darby canine kidney (MDCK) cells with *N*-tosyl-l-phenylalanine chloromethyl ketone (TPCK)-treated trypsin, which is similar to classical IAVs and different from bat H17N10 and H18N11 viruses. All eight gene segments of the H9N2-like bat isolate show a high homology to those of H7N9, H13N2, H9N2, H3N8, H7N3, and H7N1 avian influenza viruses except for the PA segment that displays a high similarity to that of an equine H7N7 virus. However, the similarity of each gene between bat H9N2-like virus and conventional IAVs is not high, ranging from 72% to 87% at nucleotide level [27]. Phylogenic analysis of its eight gene segments revealed that this H9N2-like bat virus is distinct from H17N10 and H18N11 viruses, as well as conventional IAVs, and forms a distinct lineage from known IAVs (Figure 1). All data suggest that this virus might be a very old virus and has existed in nature for a long time until its discovery. Furthermore, the virus prefers to bind to α2,3- sialic acid, not α2,6- sialic acid receptors [27]. In addition, bat H9N2-like sera can cross-react with classical avian H9N2 viruses and vice versa [27]. The cumulative evidence suggests this virus likely has an avian origin.

The H9N2-like bat virus was detected in a densely populated agricultural area and likely transmitted through the fecal-oral route similar as typical avian influenza viruses [27]. This suggests opportunities for human exposure to this kind of virus through contacting bat feces and saliva contaminated fruits. In addition, the virus was also found in another bat colony 7 km away, suggesting that the virus already disseminated to other areas [27]. Another study has shown that approximately 30% of tested serum samples collected from frugivorous bats in Ghana have antibodies that cross-react with H9 avian influenza viruses [31]. Collectively, the evidence suggests that the H9N2-like virus may exist and circulate in different bat species in different areas of the Old World.

## 3. In Vitro Virus Replication of Bat Influenza Viruses

### 3.1. Cell Lines Typically Used for IAVs Do Not Support Replication of Bat H17N10 and H18N11 Viruses

Typical cell lines such as MDCK and embryonated eggs routinely used for IAV isolation do not support bat H17N10 and H18N11 virus replication, which likely is why the viruses could not be isolated from bat samples in which viral genome sequences were identified [24,25]. To identify cell lines susceptible to bat influenza virus, pseudotyped vesicular stomatitis virus (VSV) recombinants were used to identify susceptible mammalian cell lines [32,33,34]. The pseudotype viruses expressed the HA (VSVΔG-H17, VSVΔG -H18) of bat influenza virus instead of G protein and both were found to infect canine cells (MDCK II and RIE 1495) and human cells (A549, Calu-3, 16HBE14o, SK-Mel-28, U87 MG), but only the VSVΔG -H18 was able to infect the bat EpoNi/22.1 cells [32]. Another study revealed that the VSV pseudotyped with HA and NA of bat influenza virus entered bat cells (EpoNi/22.1, HypNi/1.1, and EidNi/41), but not cells from other mammalian species [34]. Further studies demonstrated that the rescued wild type H17N10 and H18N11 viruses infected and replicated effectively on MDCK II, RIE 1495 and Calu-3 cells [32,35]. In addition, sialidase treatment increased bat virus infection and replication, and the H18N11 virus replicated more effectively under the same conditions than the H17N10 virus [32]. These facts indicate that novel bat virus HAs bind to cellular receptors for viral entry that are different from those employed by conventional IAVs.

MDCK cells are routinely used for virus isolation and amplification of conventional IAVs, but they do not support bat influenza virus replication. In contrast, bat influenza virus replicates in MCDK II cells that are derived from high passaged MDCK cells. MDCK cells have enhanced tight junctions and higher transepithelial electrical resistance than the MDCK II cells [32,36]. Conventional IAVs enter cells via the apical side of the cell membrane to initiate virus replication [37], whereas the bat influenza viruses enter cells through the basolateral membrane of polarized MDCK II cells [32,38]. Studies have shown that the functions of HA and NA of bat influenza virus are likely different from those of conventional IAVs [24,25].

### 3.2. Major Histocompatibility Complex (MHC) Class II Mediates Cell Entry of H17N10 and H18N11 Bat Influenza Viruses

HA is a glycoprotein on the surface of IAVs that is responsible for cell entry and plays a critical role in host range of IAVs. However, the HA proteins of bat influenza viruses do not have the same structure and function as those of conventional IAVs [29,39]. The head domain of HA protein is the sialic acid receptor binding pocket, which is constituted by three secondary structure loops (130-loop, 190-helix, and 220-loop) and four conserved residues (Y98, W153, H183, and Y195) [40,41,42]. In contrast, the binding region in the HA of bat influenza virus is a flat cavity which is formed by residues D136, Q190, H226, and D228 [25,39]. Due to lack of the receptor binding pocket, the HA of bat H17N10 and H18N11 influenza viruses do not bind sialic acids. Instead, the MHC class II DR protein was shown to mediate cellular entry [43,44]. The MHC class II molecule, which is constitutively expressed on the surface of professional antigen-presenting cells (e.g., macrophages, dendritic cells, B cells) and can be induced on other cells, is an important factor of T helper cell-mediated immune responses and it interacts with CD4 and the T cell receptor on those T cells [45]. Furthermore, the 293T cells co-transfected with the plasmids encoding MHC II from bats, pigs, mice, or chicken support cellular entry of the H18N11 virus [43]. Although MHC II molecules have been demonstrated to mediate both H17N10 and H18N11 virus cell entry [43,44], it remains unclear whether they are the only binding receptors or serve as necessary entry cofactors. The physical interactions between H17/H18 and MHC II molecules need to be determined to identify the binding interface in future studies.

### 3.3. Function of NA Protein of Bat Influenza Viruses Is Unclear

NA protein is the surface glycoprotein of conventional IAVs with sialidase activity that cleaves the sialic acids to release mature virus particles. In contrast to NAs of conventional IAVs, the NA of bat influenza viruses does not have sialidase activity as some important conserved amino acids at active and framework sites have changed in respective N10 and N11 [25,46,47]. Furthermore, a study has shown that bat IAV replication does not need the full-length functional NA in cell cultures by comparing virus replication between wild type H18N11 and a mutant H18N11_mut_ virus that lacks the NA head domain [48]. Additionally, the wild type H18N11 reduced the expression of MHC II on the apical membrane of MDCK cells expressing MHC II in contrast to the mutant H18N11_mut_ virus, suggesting that the bat influenza virus NA may promote virus release by down regulating the expression of MHC II molecules [48].

The replication ability is one of the important indicators of the virulence of IAVs. Bat influenza viruses can replicate in cells, but mutations are likely needed to enhance effective virus replication. One study showed that the rapid appearance of K170R and N250S substitutions on H18 head domain and the premature stop codon at G107X, which resulted in truncation of the N11 head domain, were detected after three passages of H18N11 on RIE 1495 cells, and this mutant H18N11_mut_ virus replicated much more efficiency on RIE 1495 cells than the wild type H18N11 [48]. Another study revealed that both F144C and T342A mutations of N11 protein were detected after passaging H18N11 virus on MDCK II cells, which resulted in increased virus titers in three mammalian cell lines including MDCK I, MDCK II, and human lung adenocarcinoma (Calu-3) cells [35]. These data suggest that additional mutations are required for bat IAV adaptation to cells of other host species.

## 4. In Vivo Virus Replication of Bat Influenza Viruses

Ferrets are considered to be the best small mammalian model for conventional IAVs because they are highly susceptible to infection and mirror human disease and immune response after infection [49,50]. Therefore, the ferret model is often used to assess the pathogenicity and transmissibility of conventional IAV infection in humans [49,50]. One study showed that ferrets infected with either wild type H18N11 or H18N11_mut_ did not display clinical signs of disease and appear to be healthy [48]. Replication and transmission of wild type H18N11 virus in ferrets was very low and limited because virus shedding was only detected in nasal washes collected from one of ten infected animals, and multifocal H18 RNA and weak immunoreactivity of viral matrix protein was detected in lungs of animals necropsied at early time points [48]. However, the H18N11_mut_, which has mutations in H18 head domain at K170R and N250S and the premature stop codon in N11 (G107X), replicated to detectable levels in various ferret organs such as trachea and lungs but failed to transmit to contact animals [48]. Another study also revealed that the wild type H18N11 and single NA mutated viruses (H18N11-NA-T342A or H18N11-NA-F144C) had a limited replicative ability in ferrets [35]. In contrast, the NA mutated virus possessing both the NA-F144C and NA-T342A mutations was detected in both lung and trachea of infected ferrets but none of them showed signs of disease [35]. Collectively, these results suggest that the virus with either HA or NA mutations results in a broader organ tropism in ferrets.

Similar results were observed in a mouse model and the mice did not display clinical signs of disease. Both the H18N11_mut_ and wild type H18N11 viruses infect C57BL/6 mice but they only replicated in upper respiratory airways, whereas the H18N11_mut_ virus replicated to a higher titer in upper respiratory airways than the wild type H18N11 virus [48]. Another study showed that the wild type H18N11 was able to infect BALB/c mice, but was only detected in the lung of one of three infected mice but not in upper respiratory airways such as nasal turbinate. Moreover, the single H18N11-NA-T342A or double NA mutated H18N11-NA-F144C/T342A viruses was detected in both lungs and nasal turbinates of infected BALB/c mice [35]. Both studies suggest mutations are needed to adapt to both C57BL/6 and BALB/c mice. Importantly, the MHC II-deficient mice are resistant to infection of wild type H18N11 virus, indicating that MHC II is essential for bat influenza virus infection in vivo [43].

Studies using Neotropical Jamaican fruit bats, a putative reservoir host of the H18N11 virus, show that the wild type H18N11 can infect and transmit among bats [48]. In this study, virus was detected in rectal swab samples collected from challenged and contact bats; however, virus was not detected in the lungs, suggesting that H18N11 virus is likely transmitted through the fecal-oral route similar to avian influenza viruses in their natural reservoirs, which is different from human influenza viruses through air and droplet transmission routes [51,52]. Interestingly, the H18N11_mut_ virus infected mechanically-inoculated bats but it failed to transmit to naïve contact bats. Instead, the stop codon mutation in the NA reverted to wild type in the inoculated bats, restoring the full-length neuraminidase sequence, which subsequently transmitted to contact bats. These results indicate that the fully functional N11 is required for virus transmission of H18N11 virus among bats [48]. The functions and potential mechanisms of bat influenza virus NA remain unclear, such as how the virus NA regulates expression of MHC II molecules and what is its role in virus replication and transmission, which need to be investigated.

Interestingly, the H9N2-like bat virus can replicate in lungs of both C57BL/6 and BALB/c mice even though it did not cause disease, whereas it cannot replicate in challenged domestic chickens despite its high affinity to bind α-2,3 sialic acid receptor [27]. Noticeably, this virus also cannot infect and replicate in Neotropical Jamaican fruit bats, suggesting this virus might only infect and circulate in specific bat species. Considering that the H9N2-like bat virus has not been found in other animal species so far, including birds, its biology and possibility to infect other hosts need to be investigated.

## 5. Zoonotic Potential of Bat Influenza Virus

The rapid evolution of IAVs has made a significant challenge to produce effective seasonal vaccine for humans and also contributes to the expanded host range of IAVs [21,53,54]. Gradual gene mutations and reassortment in viral genomes are two major mechanisms that are involved in the evolution of IAVs. Based on available research data, bat H17N10 and H18N11 viruses cannot replicate in human and mammalian cell lines normally used for conventional IAVs, although other cell lines have been shown to be susceptible but with rapid accumulation of mutations [32,35]. Both viruses infect ferrets and mice, but have limited replication and to a very low level [32,35]. Additionally, mutations are required to increase virus replication in both cell culture and animal models, suggesting that additional and critical mutations through a long-term evolution are needed to adapt other species. Reassortment between different subtypes or strains of IAVs is a powerful evolutionary mechanism to cross species barriers and to generate potential pandemic viruses, which led to three (1957 Asian Flu H2N2, 1968 Hong Kong flu H3N2 and 2009 pandemic H1N1) of four pandemic influenza viruses [55]. So far, available evidence shows no reassortment between bat and conventional IAVs [56,57,58] due to multiple reasons: (i) the packing signals are different in each gene segment between bat and conventional IAVs, which makes cross matching unlikely, thereby failing to incorporate the viral genome into progeny virions [56,57]; (ii) NP protein is responsible to mediate packaging eight genome segments into viral particles. Packaging process requires match between the packing code on NP and the packing signals on viral RNA packing sequence. Mismatch of the NP packing code and the packing signals between bat and conventional IAVs results in no reassortment of their genomes [59]. (iii) Recognition and replication of vRNAs by parental virus RNA-dependent RNA polymerase (RdRp), protein–protein interaction/compatibility (e.g., heterotrimeric RdRp), and vRNA–protein interactions are critical for virus reassortment. Bat influenza viruses show incompatibilities at genetic and protein levels with conventional IAVs, which cause inefficient reassortment [57]. Further studies also confirmed that reassortment between bat and conventional IAVs is impaired under traditional co-infection in cells [57,58,59]. However, reassortment occurs between H17N10 and H18N11 viruses under experimental conditions [57] and the possibility of reassortment between H17N10 and H18N11 viruses may exist in nature based on the host range of both viruses. Because H18N11 has been shown to replicate more effectively in cells than the H17N10 virus [32], reassortment between both viruses may lead to enhanced replication of the H17N10 virus. Although bat influenza viruses pose low risk for human populations [57,60], its zoonotic potential remains to be determined because they can use human MHC II molecules as an entry mediator [43]. In addition, bat influenza virus can replicate in two canine cell lines (MDCK II and RIE 1495), thus a potential route to humans could be via dogs as intermediate hosts. Conceivably, a dog could become infected if it encounters an infected bat, leading to viral genome changes that could result in transmission to its owner.

In contrast to both H17N10 and H18N11 viruses, the novel H9N2-like bat virus may pose a greater threat to humans and other species in the basis of evidence: (1) it binds to sialic acid receptors used by conventional IAVs; (2) it can be detected in peridomestic bats, resulting in transmission opportunity; (3) it can infect and replicate in mice very effectively; (4) it is likely widely distributed in bats according to available data [27,31].

## 6. Perspective

**Figure 2:**
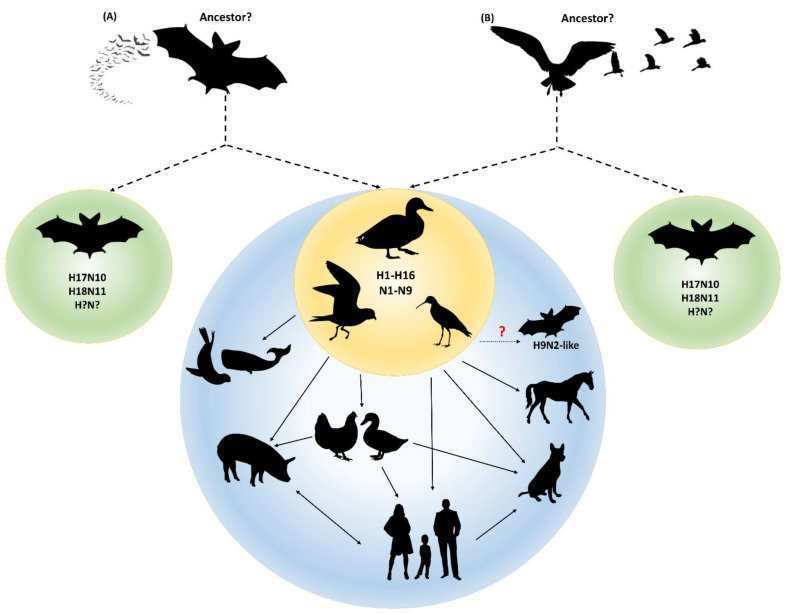
Two hypotheses on the origin of IAVs. (**A**) All IAVs originate from bats. Bats are considered to be the natural reservoirs of all IAVs that evolved for hundreds of years before transmission to birds in which rapid evolution occurred to form the classical IAVs. Some were circulating in bats to form distinct bat IAVs such as H17N10 and H18N11 (indicated by dashed arrow). (**B**) Avian species is the ancestor of all IAVs. IAVs originated from avian species in which the viruses were transmitted to wild waterfowl and bats to form classical IAVs and distinct bat IAVs (indicated by dashed arrow). Classical IAVs are circulating and evolving in waterfowl, from which they can be transmitted to a wide variety of other species. The H9N2-like bat virus is likely from the older classical IAVs that evolves very slowly in contrast to other conventional IAVs.

The discovery of bat influenza viruses changes and extends our knowledge on IAVs. Bats are the second largest group of mammals with more than 1400 recognized species. At least four species bats (*Sturnira Lilium, Artibeus planirostris*, *Artibeus obscurus*, and *Artibeus lituratus*) found in central and South America harbor bat influenza viruses [24,25,26]. Although different species of bats show different susceptibility to the same virus, frequent contact and exposure to bat influenza virus provides the opportunity to evolve and adapt in new hosts. Serological studies have shown that not only the specific bat species in which influenza viral nucleotides has been detected, but also other bat species have antibodies to either H17N10 and/or H18N11 antigens [24,25,26]. This indicates that the circulating areas and host species of bat influenza viruses likely exceeds what are currently known. To date, both H17N10 and H18N11 virus sequences as well as the novel H9N2-like virus have been detected only in bats, suggesting that bat influenza viruses continue to spread in bats and have evolved in bats for a substantial amount of time. It is possible that other novel subtypes of IAVs exist and circulate in bats, which warrants further surveillance in bat populations. In addition, an important question raised is whether IAVs originated from bats or birds. There are two hypotheses (Figure 2) on this question: (1) all IAVs originate from bats. This opinion is based on phylogenetic backdating of the internal genes which suggests that the precursor of the New World bat H17N10 and H18N11 IAV segments branched off more than 650 years ago and that the last common ancestor of Old World bat H9N2-like IAVs and conventional IAVs is around 300 years old; (2) all IAVs originate from avian species, because it is possible that an even older IAV precursor than the one found in New World bats was circulating in avian species, which was then introduced into mammals, including bats [30].

Bats are reservoir hosts many zoonotic viruses, such as the Severe acute respiratory syndrome coronaviruses, Middle East respiratory syndrome coronavirus, Nipah, Hendra, and likely Ebola viruses, which can cause severe disease and significant mortality in humans, but without clinical disease in bats [61,62,63]. Many species of bats migrate long distances, while some are found in rural and metropolitan areas, and some species are peridomestic. Due to climate change, agricultural intensification, environmental destruction, and human encroachment upon bat habitats, the potential for exposure to these pathogens is high. It is necessary to study bat ecology and virus–host interactions in order to protect public health. How bats harbor these viruses, yet do not show disease, is poorly understood. Research of these viruses in bats is necessary to understand immune responses in bats that may lead to development of countermeasures. However, many of these viruses require biosafety level 3 (BSL)-3) or BSL-4 containment, which are often not available at institutions. Bat influenza viruses are BSL-2 agents that can be a useful virus model to understand virus–host interactions and immune responses in bats.

## Data Availability

Data is contained within the article, which can be found in the cited publications.
